# Building a new research framework for social evolution: intralocus caste antagonism

**DOI:** 10.1111/brv.12394

**Published:** 2018-01-16

**Authors:** Tanya M. Pennell, Luke Holman, Edward H. Morrow, Jeremy Field

**Affiliations:** ^1^ College of Life and Environmental Sciences University of Exeter, Penryn Cornwall TR10 9FE UK; ^2^ School of Biosciences University of Melbourne Parkville Victoria 3052 Australia; ^3^ Evolution Behaviour and Environment Group, School of Life Sciences University of Sussex, Falmer East Sussex BN1 9QG UK

**Keywords:** caste antagonism, sexual antagonism, intralocus conflict, social insects, caste dimorphism, sexual dimorphism

## Abstract

The breeding and non‐breeding ‘castes’ of eusocial insects provide a striking example of role‐specific selection, where each caste maximises fitness through different morphological, behavioural and physiological trait values. Typically, queens are long‐lived egg‐layers, while workers are short‐lived, largely sterile foragers. Remarkably, the two castes are nevertheless produced by the same genome. The existence of inter‐caste genetic correlations is a neglected consequence of this shared genome, potentially hindering the evolution of caste dimorphism: alleles that increase the productivity of queens may decrease the productivity of workers and vice versa, such that each caste is prevented from reaching optimal trait values. A likely consequence of this ‘intralocus caste antagonism’ should be the maintenance of genetic variation for fitness and maladaptation within castes (termed ‘caste load’), analogous to the result of intralocus sexual antagonism. The aim of this review is to create a research framework for understanding caste antagonism, drawing in part upon conceptual similarities with sexual antagonism. By reviewing both the social insect and sexual antagonism literature, we highlight the current empirical evidence for caste antagonism, discuss social systems of interest, how antagonism might be resolved, and challenges for future research. We also introduce the idea that sexual and caste antagonism could interact, creating a three‐way antagonism over gene expression. This includes unpacking the implications of haplodiploidy for the outcome of this complex interaction.

## I. INTRODUCTION

**Figure 1:**
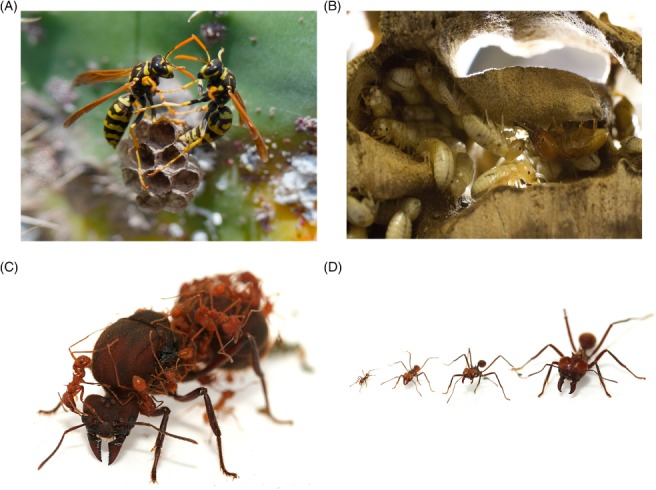
The diversity of social systems in insects. (A) Primitively eusocial paper wasps Polistes dominula: co‐foundresses fighting to attain dominance on the nest (photograph: T. Pennell). Although they are morphologically similar, the co‐foundresses will later display different behavioural phenotypes, with one female dominating most of the reproduction and the other foraging to feed the offspring. (B) Termites Pterotermes occidentis evolved sociality independently of Hymenoptera: size dimorphism shown between worker (smaller and paler) and soldier (larger and darker) castes (photograph: F. Cooney). (C, D) Advanced eusocial leaf‐cutter ants (photographs: V. Newman): (C) high reproductive skew and extreme queen and worker size dimorphism in Atta colombica; (D) size distribution of castes in Atta cephalotes, from the smallest worker caste to the largest soldier caste, known to display distinct differences in behaviour as well as morphology.

**Figure 2:**
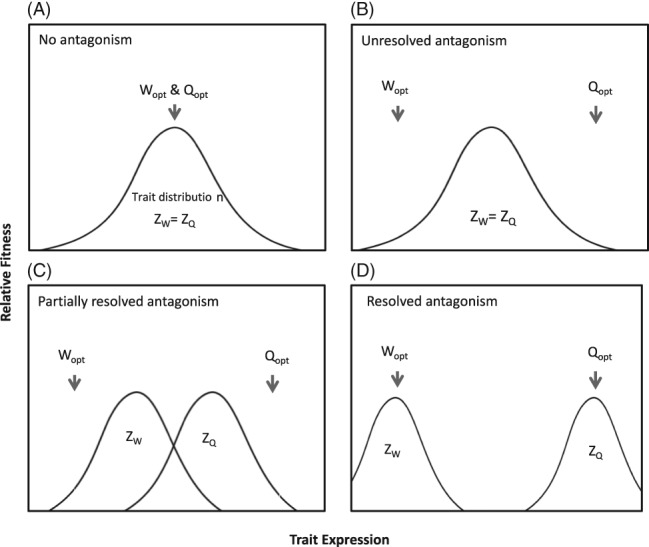
Caste dimorphism and the resolution of caste antagonism. W_opt_ and Q_opt_ (with grey arrows) represent optimal fitness values for a quantitative trait in workers and queens, respectively. The phenotypic distribution of this trait in workers (Z_W_) and queens (Z_Q_) is shown by the curve. (A) No caste antagonism: workers and queens have the same optimal trait value and the mean of the trait distribution in the population matches this value. (B) Workers and queens have different optimal values for the same trait; however there is no caste dimorphism. In this example, both castes suffer fitness loss, as they are not at their respective optima. (C) Partially resolved antagonism: partial caste dimorphism has allowed each caste to approach its fitness optimum. (D) Fully resolved antagonism: worker and queen phenotypic distributions match their respective fitness optima. Figure adapted from Cox & Calsbeek (2009).

Individuals within a population often take different routes to maximise fitness, which they follow by having distinct morphological, behavioural and physiological trait values. Familiar examples include males and females (Trivers, 1972; Bonduriansky & Chenoweth, 2009), as well as the fighter/sneaker dimorphisms displayed by some male invertebrates (Morris *et al*., 2013). The breeding and non‐breeding ‘castes’ of eusocial and cooperatively breeding animals provide a particularly striking case where role‐specific selection operates. In eusocial taxa, workers sacrifice their own reproduction to aid the reproduction of queens (Hamilton, 1964): queens are long‐lived and fecund, but workers are typically short‐lived foragers (Keller & Genoud, 1997). While social insects include the most extreme examples of specialisation for reproductive altruism (Fig. 1), the shared genetics of queens and workers has the potential to constrain their independent adaptation even in the face of divergent selection for different roles (Fig. 2; Linksvayer & Wade, 2005; Pennell & Morrow, 2013; Holman, Linksvayer & D'ettorre, 2013; Holman, 2014). This could occur whenever the fittest allele at a given locus is not the same across the different castes. This concept, hereafter referred to as ‘intralocus caste antagonism’ or ‘caste antagonism’, is characterised by antagonistic selection between castes coupled with an inter‐caste genetic correlation, such that selection in one caste causes a correlated response in the other caste. For example, an allele causing greater investment in ovary development might be advantageous for queens, but the same allele might reduce the efficiency and productivity of workers by making resources unavailable for tasks such as foraging. A likely consequence of caste antagonism is the maintenance of genetic variation within both castes for fitness‐related traits, leading to maladaptation and loss of fitness (Holman, 2014), thereby generating a ‘caste‐load’. Note that while we use ‘caste antagonism’ and ‘caste conflict’ interchangeably in this review, the word ‘conflict’ has often been used in a somewhat different sense in the social insect literature, to indicate that individuals in a social group have different fitness interests. For example, queens are usually said to be ‘in conflict’ with workers when their evolutionary interests are best served by a more female‐biased sex investment ratio than the ratio preferred by workers (Ratnieks & Reeve, 1992).

Intralocus antagonism was a concept initially developed in relation to the antagonism between males and females. Analogous to caste antagonism, ‘sexual antagonism’ is defined by a negative inter‐sexual genetic correlation for fitness, where individuals of one sex with high fitness tend to have relatives of the opposite sex with low fitness. This conflict arises because the divergent reproductive roles of the two sexes favour sex‐specific phenotypes (Trivers, 1972; Parker, 1979), but their shared genome places limits on the degree of sexual dimorphism that can evolve (Lande, 1980). Because of the clear analogies between caste antagonism and sexual antagonism, we believe that research relating to one conflict can aid our understanding of the other, a link not yet fully exploited by researchers in either field. However, because the two conflicts can arise in very different biological contexts, they are also interesting to compare. Herein, we will characterise caste antagonism, examine how genetic, ecological and social differences set it apart from sexual antagonism, and discuss the extent to which the same mechanisms used to resolve one conflict may be deployed to resolve the other. Additionally, we suggest that caste antagonism and sexual antagonism will act simultaneously to constrain trait evolution in social species, with interesting and unexplored consequences. We will use social insects as examples in our review, but many of the same ideas could apply to other social taxa such as cooperatively breeding vertebrates. Currently, our understanding of sexual antagonism is well developed, with both theoretical (Rice, 1984; Day & Bonduriansky, 2004; Connallon, Cox & Calsbeek, 2010; Connallon & Clark, 2012, 2014b) and empirical evidence suggesting its widespread occurrence in non‐social sexual taxa (reviewed in Bonduriansky & Chenoweth, 2009; Van Doorn, 2009; Pennell & Morrow, 2013). Caste antagonism should be similarly ubiquitous in social systems (Holman, 2014), but is much less well studied. Our ideas about caste antagonism are therefore necessarily speculative, but by interrogating both the social insect and sexual antagonism literature, we aim to highlight exciting new directions for research in social taxa.

## II. INTER‐CASTE GENETIC CORRELATIONS WILL LEAD TO CASTE ANTAGONISM

Given that the optimal phenotypes of different castes are unlikely to be identical, their shared genomes could mean that selection on one caste will lead to a maladaptive correlated response in the other. The potential result is a negative correlation between the fitness effect of an allele when expressed in a breeder *versus* a non‐breeder – the hallmark of caste antagonism. Although inter‐caste genetic correlations for fitness itself have not yet been measured in any social system, inter‐caste genetic correlations for fitness‐related traits have been identified that are indicative of caste antagonism. For example, a positive inter‐caste genetic correlation for ovarian development was found in the ant *Lasius niger*, such that especially fecund queens tended to produce more fecund workers (Holman *et al*., 2013). High fecundity is hypothesised to be beneficial for queens, but in workers could be detrimental for colony productivity, since it might direct resources away from worker‐specific tasks such as foraging (Franks & Scovell, 1983). Specifically, worker ovary development could be deleterious if it reduces the indirect fitness that workers could gain through helping their relatives to reproduce. Additionally, Holman (2014) calculated that around 134 RNA transcripts appear to affect honeybee *Apis mellifera* queen and worker fecundity pleiotropically in the same direction, based on microarray data from brain tissue (Grozinger *et al*., 2007). A study of bumblebees *Bombus terrestris* also found that reproductive workers and queens had more similar gene expression profiles than did reproductive and non‐reproductive workers (Harrison, Hammond & Mallon, 2015), again suggesting pleiotropy across castes (as well as some caste‐specific expression) for genes affecting fecundity. Measuring the inter‐caste correlation for fitness in social taxa could be challenging due to the difficulties of disentangling genetic and environmental effects in social groups and quantifying caste‐specific fitness, but it is nonetheless feasible (see Table 1 and Section V). In sexual conflict research, negative inter‐sexual genetic correlations for total fitness have been demonstrated in a wide range of taxa, including insects (Chippindale, Gibson & Rice, 2001; Rice & Chippindale, 2001; Gibson, Chippindale & Rice, 2002; Bonduriansky & Rowe, 2005a, 2005b; Pischedda & Chippindale, 2006; Long & Rice, 2007; Bedhomme *et al*., 2008; Harano *et al*., 2010; Innocenti & Morrow, 2010; Hesketh, Fowler, & Reuter, 2013; Berger *et al*., 2014), birds (Tarka *et al*., 2014), reptiles (Svensson, McAdam & Sinervo, 2009), humans (Garver‐Apgar *et al*., 2011; Stulp *et al*., 2012) and other mammals (Mainguy *et al*., 2009; Mills, Koskela & Mappes, 2012; Mokkonen *et al*., 2012). These examples show that a shared genome can constrain all of the parties involved across biologically diverse systems. Such constraints are likely to affect any individuals that share the same genes but have different optimal phenotypes; including, but not limited to, the social castes.

**Table 1:** Possible approaches to the investigation of caste antagonism, including different approaches that could be used to research caste antagonism in social systems, and analogous methods that have been used successfully in the study of sexual antagonism.

Approaches for caste antagonism	Parallels in sexual antagonism
**Estimate additive effects of genes on caste phenotype and inter‐caste genetic correlations using:**	
**(1) Cross‐fostering**. Offspring are raised by surrogates, to disentangle genetic and environmental effects on phenotype. This method has been used successfully in ants to identify the genetic basis of worker and gyne mass, caste ratio Linksvayer (2006) and worker size Linksvayer (2007) and to investigate the genetic correlation between queen and worker fertility and hydrocarbons Holman *et al*. (2013). In bees, cross‐fostering was employed to determine the effects of social experience on aggression Rittschof *et al*.(2015) and body/ovary size Linksvayer *et al*.(2009). Nest‐mate recognition in wasps was also determined by cross‐fostering in the wild Costanzi *et al*.(2013).	Cross‐fostering is especially common in birds, where it has been used to the estimate inter‐sexual genetic correlations for facial pattern Potti & Canal (2011), testosterone levels Iserbyt, Eens & Müller (2015) and plumage colouration Drobniak *et al*.(2013).
**(2) Full‐sib breeding designs.** Quantitative measurement of traits in full‐sibs (brothers and sisters) to tease apart environmental and genetic components of these traits. Controlled crosses used for breeding designs are possible in social insects, including ants Schwander & Keller (2008) and Frohschammer & Heinze (2009) and termites Hayashi *et al*.(2007). These have commonly been used to explore genetics effects on caste determination.	Used in beetles to show inter‐sexual correlation for same‐sex mating behaviour Burgevin, Friberg & Maklakov (2013) and for multiple sexually dimorphic traits in flies Bonduriansky & Rowe, (2005a).
**(3) Inbred lines**. Samples from a population that has been subject to inbreeding over multiple generations, leading to homozygosity. It is then possible to explore the phenotypes of different individuals that share near‐identical genotypes. Some social animals naturally show high levels of inbreeding, making them well suited to this approach. For example, inbred lines have been used in bees and ants to test hypotheses regarding odour recognition Greenberg (1979) and Mintzer & Vinson (1985). General effects of heterozygosity level on developmental stability have also been investigated in bees using inbreeding Clarke *et al*.(1992).	Isofemale lines were used to measure inter‐sexual genetic correlations for fitness across environments [in beetles Berger *et al*.(2014) and flies Punzalan *et al*.(2014)]. Also used in crickets to demonstrate inter‐sexual genetic correlations for ageing and lifespan (Archer *et al*., 2012).
**(4) Animal models**. By genotyping a wild population to infer relatedness among many individuals, one could use mixed models to estimate genetic covariance between castes. Microsatellite genotyping is a useful method for analysing pedigrees [for examples in wasps and ants see Queller *et al*. (2000), Leniaud *et al*. (2013) and Huang *et al*. (2013)].	Wild‐pedigreed populations of deer Foerster *et al*. (2007) sheep Poissant *et al*.(2008), lizards Svensson *et al*.(2009) and birds Brommer *et al*.(2007) used to calculate inter‐sexual genetic correlations for fitness.
**(5) Patrilines (clonal sperm)**. Fertilizing eggs from different females with genetically identical sperm from hymenopteran males produces individuals of the same haplotype expressed in different genetic backgrounds. Can be used to explore the additive effects of genes when expressed in a heterozygous state. Examples of patriline experiments in social insects include studies of ants showing a genetic component to caste determination Hughes *et al*.(2003) and Smith *et al*.(2008) and disease resistance Hughes & Boomsma (2004), and research in bees showing a genetic basis to worker behaviour Kraus *et al*.(2011).	Hemiclonal analysis is analogous to fertilizing different eggs with clonal sperm. Instead of relying on a naturally occurring system, genetic tools available in the fly model, including balancer and fused‐X chromosomes, force the inheritance of whole, intact haplotypes. One can create large numbers of individuals of both sexes that share the same haplotype. This method has been used in flies to show inter‐sexual genetic correlations for fitness Chippindale *et al*.(2001), Innocenti & Morrow (2010).
**Identify genes underlying caste antagonism** Correlating allelic variation, or gene expression, with caste‐specific fitness. This will help to uncover the molecular basis of traits underlying antagonism. It will also aid our understanding of the genetic constraints that prevent complete caste‐specific expression, e.g. pleiotropic and epistatic constraints.	The involvement of alleles associated with existing antagonism Rostant *et al*.(2015) and Hill *et al*.(2017)) and its resolution Collet *et al*.(2016) has been shown in fly populations. There is also an understanding of genome‐wide patterns of gene expression that contribute to sexual antagonism in fly populations Innocenti & Morrow (2010) and Ingleby *et al*.(2016).

## III. THE RESOLUTION OF CASTE ANTAGONISM

### (1) Caste dimorphism

Caste‐specific selection selects for divergent trait values, suggesting that caste dimorphism can represent at least partially resolved caste antagonism (Fig. 2; Holman, 2014). Here, we discuss evidence for caste‐specific selection and the possible genetic mechanisms through which caste dimorphism could arise.

Caste dimorphism is likely to be affected by caste‐specific differences in the expression level of particular genes, including where and when these genes are expressed in the body. In support of this, castes differ in many aspects of their behaviour, morphology and physiology, particularly in ‘advanced’ eusocial lineages with large, perennial colonies and specialised social adaptations, and these phenotypic differences are accompanied by substantial inter‐caste differences in gene expression (e.g. Ferreira *et al*., 2013; Simola *et al*., 2013; Feldmeyer, Elsner & Foitzik, 2014; Harrison *et al*., 2015; Morandin *et al*., 2015). Common functions associated with genes showing caste‐specific expression include reproduction (egg production), metabolism, somatic maintenance and repair, digestion and feeding, pheromone recognition, cellular activity, protein structure and immunity, as well as many novel genes of unknown function (Ferreira *et al*., 2013). These extensive transcriptomic differences are consistent with caste‐specific selection across much of the genome. These differences also suggest that a conserved set of genes might be responsible for the evolution of sociality across species (Toth & Robinson, 2007). Mechanisms underlying gene expression differences, such as gene duplication, DNA methylation and alternative splicing, will be discussed in more detail in Section III.2. Caste‐biased patterns of gene expression might also arise *via* neutral processes. However, neutrality could be difficult to demonstrate (Helanterä & Uller, 2014), and neutral and antagonistic processes could operate together.

### (2) Mechanisms facilitating caste dimorphism

Like caste dimorphism, sexual dimorphism can represent past or ongoing intralocus antagonism (Cox & Calsbeek, 2009). It is therefore likely that mechanisms facilitating sexual dimorphism (Stewart, Pischedda & Rice, 2010) play similar roles in the differentiation of castes. The same mechanisms are also relevant to other polyphenisms besides sex and caste, such as alternative reproductive tactics that can occur within a sex (Morris *et al*., 2013). One key mechanism identified is gene duplication followed by sub‐functionalisation (i.e. the acquisition of a novel function by one of the resulting pair of genes), which has been hypothesised to play a role in both mitigating sexual antagonism (Gallach & Betrán, 2011; but see Hosken, 2011) and producing caste‐specific gene expression (Claudianos *et al*., 2006; Xu *et al*., 2010, Terrapon *et al*., 2014). This could involve the movement of an antagonistic allele to a genomic location that is already under caste‐specific regulation, or to where caste‐specific regulation might subsequently evolve. Some vitellogenin (*Vg*) genes that are linked to egg production and caste determination are recent duplicates that differ in level of expression between queens and workers (Terrapon *et al*., 2014). The same is true for hexamerins, which play a role in soldier caste development (Terrapon *et al*., 2014), and the heat shock protein 90 (HSP90) stress protein that is differentially expressed between castes (Xu *et al*., 2010).

DNA methylation, and other epigenetic mechanisms that affect gene expression also have the potential to mediate polyphenisms such as caste dimorphism. This could work to alleviate caste conflict if the choice of methylated allele was dependent on caste. Many of the social Hymenoptera possess a full set of genes for applying, maintaining, and responding to DNA methylation (Patalano *et al*., 2015). In adult *A. mellifera*, some studies suggest that the methylome is caste‐specific (Lyko *et al*., 2010; Foret *et al*., 2012), although better‐replicated studies in *A. mellifera* (Herb *et al*., 2012) and the paper wasp *Polistes canadensis* (Patalano *et al*., 2015) found no caste‐specificity. Nevertheless, in *A. mellifera* knockout of a DNA methyltransferase gene (*dnmt3*) caused worker‐destined larvae to develop queen‐like traits (Kucharski *et al*., 2008) and significantly affected gene expression for 17% of the transcriptome (Li‐Byarlay *et al*., 2013), consistent with a role for methylation in mediating polyphenism. In *B. terrestris*, workers treated with a DNA de‐methylation agent developed queen‐like traits, and there is support for differential methylation between reproductive and non‐reproductive workers (Amarasinghe, Clayton & Mallon, 2014). In the ant species *Camponotus floridanus* and *Harpegnathos saltator* DNA methylation is again thought to be caste‐specific (Bonasio *et al*., 2012). However, in line with bee and wasp research, experiments with greater replication in the ants *Dinoponera quadriceps* (Patalano *et al*., 2015) and *Cerapachys biroi* found no evidence of caste‐specificity (Libbrecht *et al*., 2016). Termites, a lineage where sociality has evolved independently of Hymenoptera, also have DNA methylation, and there is some evidence that it may similarly encode differences between castes in *Reticulitermes flavipes* and *Coptotermes formosanus* (Glastad, Hunt & Goodisman, 2013). Furthermore, histone modifications, another important type of epigenetic modification, have been found to differ between castes in the ant *C. floridanus* (Simola *et al*., 2013). Analogously, sex‐specific methylation is a potential mechanism underlying sexual dimorphism (El‐Maarri *et al*., 2007; Avila *et al*., 2010; Bermejo‐Alvarez *et al*., 2010; Liu *et al*., 2010; Xu *et al*., 2013; Hall *et al*., 2014), and the case for its involvement in alleviating conflict is particularly strong. Methylation is identified predominantly on the X chromosome (Xu *et al*., 2013), which is predicted to be enriched for sexually antagonistic alleles (Gibson *et al*., 2002; Lindholm & Breden, 2002; Fitzpatrick, 2004; Tower, 2006; Innocenti & Morrow, 2010), and there is evidence that Y‐linked histone methylation modulates autosomal gene expression (Lemos, Branco & Hartl, 2010), which is a possible route to sex‐specific expression.

Genomic imprinting, which is sometimes mediated by DNA methylation, could also theoretically promote adaptive caste dimorphism, mirroring an interesting hypothesis from the sexual conflict literature (Day & Bonduriansky, 2004). In one iteration of that hypothesis, females might benefit by expressing only the allele they inherited from their mother (at one or many loci), and males might benefit by only expressing their paternally derived allele; this hypothesis has received some support (Hager *et al*., 2008), but as yet has not received much study. Although beyond the scope of this review, formal models are needed to confirm how this hypothesis would work under caste antagonism, because of complications resulting from the simultaneous operation of caste and sexual antagonism (see Section IV.1). For example, workers might benefit from silencing the queen‐derived allele, if the average allele derived from the queen's mate is closer to the worker optimum than the average queen‐derived allele. However, if male‐derived alleles confer a worse worker phenotype than queen‐derived alleles, it might pay workers to silence their paternally derived allele. A recent study of *A. mellifera* found evidence of parent‐of‐origin‐specific gene expression (i.e. genomic imprinting: Galbraith *et al*., 2016); specifically, paternally derived alleles were more highly expressed than maternal alleles in worker reproductive tissues. The authors correctly interpreted this result as being consistent with an alternative theory of imprinting (the ‘kinship theory’), but another possible evolutionary explanation for this result is that workers are expressing the male‐derived allele because male‐derived alleles are (on average) closer to the worker phenotypic optimum than queen‐derived alleles are (particularly since the ovary is a tissue experiencing strong caste antagonism). Evidence of allele‐specific DNA methylation was also found in the ants *C. floridanus* and *H. saltator*, consistent with preferential methylation of one parent's allele (Bonasio *et al*., 2012). Additionally, the allele that was methylated for some loci was different in queens and workers, hinting at the possibility of caste‐specific genomic imprinting, in which offspring that are workers methylate one parent's allele and those that are queens methylate the other. This would be consistent with our suggestion that queens could maximise their fitness by expressing only the queen‐derived allele and workers by expressing the male‐derived allele, similar to the proposal by Day & Bonduriansky (2004) under sexual antagonism.

Imprinting could also work by mediating other forms of gene regulation such as alternative splicing (Li‐Byarlay *et al*., 2013), where different protein forms are produced from the same gene. This process has been linked to sex‐specific polyphenism (Telonis‐Scott *et al*., 2009; Brown *et al*., 2014; Ingleby *et al*., 2016) and evidence is accumulating for widespread caste‐specific alternative splicing (Aamodt, 2008; Jarosch *et al*., 2011; Bonasio *et al*., 2012; Foret *et al*., 2012; Terrapon *et al*., 2014). The association between these two mechanisms can be seen by examining protein isoforms at particular genomic locations. For example, DNA methylation tends to occur at CpG sites (cytosines followed by guanine residues), and these sites appear to co‐occur with alternative splice events (Terrapon *et al*., 2014). This link suggests the preferential targeting of alternatively spliced genes by DNA methylation.

Despite similarities in the mechanisms through which caste and sexual antagonism might become resolved, there are also likely to be differences. In sexually dimorphic species that have chromosomal sex‐determination, sexual antagonism can potentially be mitigated through the movement of strongly sexually antagonistic alleles from autosomes to sex chromosomes. For example, sexually antagonistic alleles (or regulatory elements that affect their expression), which benefit only the heterogametic sex might be selected to move to the Y (or W) chromosome, resulting in adaptive, sex‐limited expression (Rice, 1984). Sex chromosomes can be thought of as ‘supergenes’ because they are tight clusters of loci affecting developmental characteristics. There is evidence of a similar ‘supergene’ in the fire ant *Solenopsis invicta*, which is a non‐recombining autosomal region with two variant chromosome forms. These chromosomes underlie social phenotypes and contain many loci that are associated with life‐history differences among individuals (queens, workers and males) that carry the alternate forms (Wang *et al*., 2013). Like sex chromosomes, it seems probable that the *S. invicta* supergene arose due to antagonistic selection favouring reduced recombination to give rise to different adaptive phenotypes. Although this supergene can control phenotype within a caste or sex, there is no evidence for its involvement in caste or sex determination. In fact, a purely genetically determined caste‐determination system such as a supergene might not evolve because kin selection requires workers and queens to both carry alleles that give them the potential to reproduce; moreover, genetic caste‐determination might cause developmental constraint, leading to non‐optimal resource allocation [see Schwander *et al*. (2010) for a review]. Given that hymenopteran queens and workers share identical chromosomal complements and that evidence of supergenes in social insects is rare (Wang *et al*., 2013; Purcell *et al*., 2014), it follows that methylation or alternative splicing may be the most important mechanisms of resolution in Hymenoptera.

Caste polymorphism has evolved multiple times within individual clades (e.g. eight times within Hymenoptera; Hughes *et al*., 2008), and the same mechanisms, such as DNA methylation and alternative splicing, seem to have been implicated in mediating caste polyphenism across multiple independent evolutionary origins [e.g. termites (Glastad *et al*., 2013; Terrapon *et al*., 2014); ants (Bonasio *et al*., 2012); bees (Aamodt, 2008; Lyko *et al*., 2010; Foret *et al*., 2012)]. This similarity could reflect convergent evolution, but it also seems likely that the evolution of sociality involved the repeated co‐option of evolutionarily ancient mechanisms for regulating gene expression (see Klein *et al*., 2016). The latter possibility is consistent with the ‘theory of facilitated variation’ (Kirschner & Gerhart, 1998), whereby ancient regulatory genes with large, relatively conserved sets of downstream targets are postulated to be the main sources of evolutionary novelty. For example, castes might have arisen when a regulatory gene responsible for stimulating transcription of genes involved in oogenesis, perhaps in response to cues indicating developmental maturity, began responding instead to the level of larval nutrition (Rehan & Toth, 2015). Variation in larval nutrition could then begin to determine caste. An attractive feature of this hypothesis is that it explains how the evolution of a radical new trait such as caste dimorphism can arise spontaneously *via* simple modifications to pre‐existing systems (see Klein *et al*., 2016), such that the new phenotype is coordinated and functionally integrated in a way that would be improbable under alternative evolutionary scenarios (e.g. sequential fixation of various disparate novel mutations). *Vg* might be an example of a gene that has been co‐opted for caste differentiation, although direct evidence of its role during caste development is lacking. *Vg* is highly pleiotropic: it is involved in egg production and has recently been shown to play a role in sexual behaviour in a subsocial beetle (Roy‐Zokan *et al*., 2015), but it has also been linked to behavioural changes associated with the reproductive division of labour between castes (Nelson *et al*., 2007; Amdam & Page, 2010). *Vg* also shows an antagonistic relationship with juvenile hormone in many insects (Fluri *et al*., 1981; Fahrbach, Giray & Robinson, 1995; Corona *et al*., 2007), a major endocrine effector that shows caste‐specific activity and which has a complex relationship with fecundity (Holman, 2012). Intriguingly, other genes that are involved in sexual dimorphism, such as the ancient sex‐differentiation genes *doublesex* and *transformer*, appear also to regulate other polymorphisms, including queen–worker dimorphism (Klein *et al*., 2016), the development of large‐ and small‐horned male morphs in a dung beetle (Gotoh *et al*., 2014), and butterfly wing colouration (Kunte *et al*., 2014). Similarly, the sex differentiation gene *fem* is also expressed differentially between castes in a stingless bee (Brito *et al*., 2015).

### (3) Association between caste dimorphism and resolution of conflict

Although caste conflict can lead to caste dimorphism, the use of caste dimorphism as a proximate measure of caste conflict across species is problematic. This is because the relationship between caste dimorphism and conflict is not simply positive or negative, as the genetic architecture of caste‐specific traits and the nature of caste‐specific selection can change in complex ways as dimorphism evolves.

Despite this complex relationship, caste polymorphism can mitigate intralocus caste antagonism over specific phenotypic traits within a population, just as sexual dimorphism can reduce intralocus sexual antagonism (Fig. 2; Cox & Calsbeek, 2009). For example, within a social insect population a colony is likely to perform better if the castes are more specialised in their roles, and hence more dimorphic. In a similar way, higher population fitness is associated with greater sexual dimorphism in beetle populations [size dimorphism (Rankin & Arnqvist, 2008) and dimorphism in development time (Arnqvist & Tuda, 2009)]. Nonetheless, empirical and theoretical research (e.g. Innocenti & Morrow, 2010; Connallon *et al*., 2010; Connallon & Clark, 2014b) also suggests that intralocus sexual antagonism is rarely (if ever) completely removed by the evolution of dimorphism, and we expect the same to be true of caste antagonism.

Studies of sexual antagonism suggest that multiple factors will conspire to make the evolution of caste dimorphism incomplete, such that caste antagonism will often be strong in practice. For example, high standing levels of caste antagonism might result from pleiotropic and epistatic constraints on the evolution of caste‐specific gene regulation [see Van Doorn (2009), Pennell & Morrow (2013) and Connallon & Clark (2014a) for sexual antagonism]. Recent research on 15 ant species suggests that this is likely to be true, as within species gene connectivity was negatively correlated with the extent of caste dimorphism in gene expression (Morandin *et al*., 2017). Additionally, spatial/temporal variation in selection could help to maintain caste antagonism by creating inconsistent selection for caste‐biased gene expression, such that it does not evolve (see Pennell & Morrow, 2013; Pennell *et al*., 2016). Although recent research has illustrated how mutational pleiotropy (Holman, 2014) and gene connectivity (Morandin *et al*., 2017) could prevent the resolution of caste antagonism, no other barriers to caste‐conflict resolution have yet been explored empirically or theoretically. To date, genomic and transcriptomic research on social insects has focused mostly on the mechanisms that produce phenotypic differences between castes (e.g. by searching for differences in gene expression or DNA methylation between castes), but not on the role that these mechanisms may have in alleviating caste antagonism – the implicit assumption being that queens and workers have already achieved perfect ‘genetic release’ (Gadagkar, 1997), and are expressing an optimal caste‐specific phenotype. However, as discussed earlier, there is evidence for unresolved caste antagonism despite the occurrence of dimorphism (see Holman *et al*., 2013; Holman, 2014). Research has also focussed on expression differences in adults and not on developmental stages, where caste‐specific selection might be strongest. Genetic correlations between different life stages are also expected to be rife, both within and among castes (Ingleby *et al*., 2016). Such complex interactions deserve further attention as barriers to conflict resolution. The same framework could also be applied to alternative reproductive morphs within a sex or caste, where developmental constraints on polyphenism might arise.

## IV. A THREE‐WAY ANTAGONISM

### (1) Selection on males

**Figure 3:**
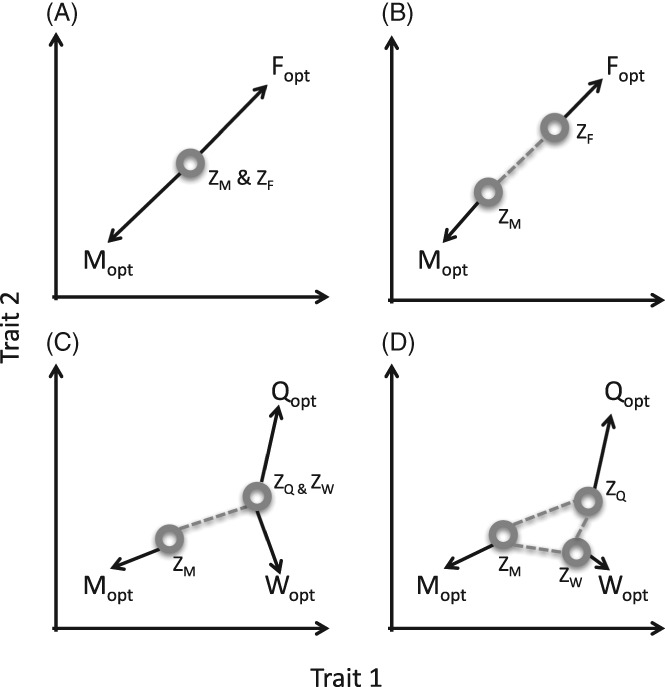
Interactions between sexual antagonism and caste antagonism. Sexual antagonism and caste antagonism should act in concert to shape the phenotype of dioecious, social species. Symbols indicate current phenotypes of males (Z_M_), females (Z_F_), queens (Z_Q_) and workers (Z_W_); unconstrained optimum phenotypes are indicated with ‘opt’ subscripts. (A) Sex‐specific selection is shown by black lines with arrowheads representing the directions of selection pulling a sexually monomorphic phenotype (the grey circle) towards divergent optima (M_opt_ for males and F_opt_ for females). (B) Sexual dimorphism has evolved but genetic correlations between the sexes (shown by dashed grey line) prevent the male and female phenotypes from reaching their optima. (C) The species has evolved queen and worker castes (optimal phenotype represented by Q_opt_ and W_opt_, respectively), but there is not yet any phenotypic divergence between castes. However, the phenotype of both males and queens is deflected by selection on workers. (D) The species has evolved caste dimorphism, but maladaptation remains because of genetic correlations between sexes and castes. In this example, queens have become more maladapted than males because of worker‐specific selection. The evolutionary outcome can be thought of as a tug of war: the positions of the three phenotypes in multivariate space depend on the strength and efficiency of selection pulling the shared phenotype towards three different optima, and on the extent of genetic constraints that prevent the phenotypes from complete divergence.

Traits in many male Hymenoptera are presumably under strong selection, especially in species with male‐biased operational sex ratios. Given that males obtain fitness through routes that are very different from either queens or workers, we suggest that sexual antagonism and caste antagonism will act simultaneously in dioecious social species. This additional complexity occurs because the genetic architecture that is common to queens and workers is also shared with males. The net evolution of a shared trait will therefore depend on its fitness consequences when expressed in different sexes as well as castes, and we predict that selection will frequently fail to optimise the fitness of all three phenotypes (Fig. 3). Often, males, queens and workers will all have distinct mean values for a shared trait, implying that each has a different optimum and that both sexual antagonism and caste antagonism are only partially resolved (Cox & Calsbeek, 2009). For example, males, queens and workers are commonly distinct in terms of body size, morphology and physiology (Stubblefield & Seger, 1994; Hrassnigg & Crailsheim, 2005; Zayed & Kent, 2015). In other cases, queen and worker trait values are similar but differ greatly from those of males; for example, *L. niger* males have a very short lifespan and also short telomeres relative to queens and workers, while the female castes differ in lifespan but not telomere length (Jemielity *et al*., 2007). For other traits, reproductives (queens and males) differ from workers, for example in wing phenotype (e.g. in ants: Abouheif & Wray, 2002) and gamete production. In short, it seems certain that some loci are under both sexual antagonism and caste antagonism, while selection at other loci may be concordant across some sexes/castes but not others. Knowledge of which genes undergo sex‐biased expression would provide a better idea of sex‐specific selection. The molecular basis of sexual dimorphism has been largely unstudied in Hymenoptera, with some exceptions (e.g. Nipitwattanaphon *et al*., 2014; Schrader *et al*., 2015; Schrader, Helanterä & Oettler, 2016).

Males are understudied in social insects, perhaps because they are rarely involved in parental care, are present for only part of the colony lifecycle, and because mating is often difficult to observe. There is also a tendency to regard sexual selection as comparatively weak and free of antagonism in the social insects, with males described as having ‘few if any sexually selected traits’ (Boomsma, Baer & Heinze, 2005, p. 396). However, a male's ability to reach maturity, search for females, mate, and fertilise eggs is presumably highly polygenic, so that much of the genome may contribute to variance in reproductive success, and thus by definition be under sexual selection (*cf*. Rowe & Houle, 1996; Whitlock & Agrawal, 2009). Moreover, social insect colonies often produce many more males than queens, because males are cheaper to produce than queens (Charnov, 1982). This potentially creates large variance in male fitness, and hence strong selection on traits that affect male mating success. Together, these factors suggest that selection on males might affect much of the genome, even in monogamous species lacking ‘active’ sexual selection (choosy females, male fighting, etc.) or classically sexually selected adaptations such as ornaments and weapons. A unique case of hybridisation between two *Formica* ant lineages showcases how selection on males can dramatically alter genetic architecture within a species. When the two lineages interbreed, the surviving female offspring have a hybrid genetic background, whereas the surviving males do not. This difference is due to strong selection against males carrying the hybrid genotype (Kulmuni & Pamilo, 2014).

An additional complexity is that male Hymenoptera are haploid while females (i.e. queens and workers) are diploid, and so all alleles are fully exposed to selection in males, while in females recessive alleles can ‘hide’ from selection in heterozygotes. Haplodiploidy thus causes a difference in the efficacy of selection between the sexes, which might skew the average phenotype towards the male optimum. The *Formica* ant hybridisation discussed above may result from male haploidy, whereby selection on males purges alleles from the other lineage but selection on females does not, because the fitness effects of being a hybrid are recessive (Kulmuni & Pamilo, 2014). This is not the case in other social insects, such as termites, where both males and females are diploid.

The outcome of caste antagonism should also depend on the extent to which each caste relies on direct *versus* indirect transmission of its genes. Selection on worker traits becomes weaker (relative to selection on queen traits) when workers gain little or no fitness from direct reproduction, and when the average relatedness between workers and the recipients of their help is low (see Van Dyken, Linksvayer & Wade, 2011). This means that species with sterile workers, and species in which workers care for individuals to whom they are not closely related, should have a caste‐specific phenotype that is closer to the queen optimum (Hall, Yi & Goodisman, 2013; Holman, 2014). Additionally, the fitness of an individual is influenced not only by the direct effects of its own genotype, but also by the genotype of its social partners. For example, the direct fitness of a male will be depend on whether the genes he carries are well adapted in males, but it will also be influenced indirectly by whether his social partners (e.g. the workers that raised him) carried genes that suited their own roles. Evolutionary predictions are further complicated when direct and indirect genetic effects such as these covary (e.g. because social partners are kin), and this can strongly deflect evolutionary trajectories in unexpected directions (McGlothlin *et al*., 2010). Formal models are clearly required for a proper understanding of caste antagonism.

### (2) Other sources of antagonistic selection acting in social insects

The existence of multiple sub‐castes, such as different size categories of workers, will also affect the outcome of the tug‐of‐war over gene expression. This will be influenced by their specific roles in the colony and the selection pressures that they experience. For example, *Pheidole* ants are categorised into minor and major workers, with minor workers taking on general ‘housekeeping’ duties within the nest, and major workers specialising in colony defence, food storage and seed milling (Wilson, 2003).

Furthermore, Morris *et al*. (2013) proposed that in species with alternative reproductive tactics (e.g. sneaker and fighter males), selection may act antagonistically on shared traits of the two morphs (they termed this ‘intralocus tactical conflict’). *Cardiocondyla obscurior* ants produce multiple types of males: winged dispersive males, and wingless ‘ergatoid’ males that are specialised for fighting and mate in their natal nest (Klein *et al*., 2016). This species effectively has four body plans to build with its single genome (queens, workers, and two kinds of males), and intriguingly there is evidence that similar genetic mechanisms may control caste differentiation in males and females (Klein *et al*., 2016).

Reproductive females might also be sub‐categorised in the same manner. Several ant species produce both large queens, capable of founding new colonies alone, and small queens, which either join existing colonies or found new colonies by ‘budding’ with a group of workers (Rueppell & Heinze, 1999). In the *S. invicta* example discussed previously, queen morphology and life history is genetically determined, whereby queens of different genotypes differ in odour, fat accumulation during sexual maturation, and fecundity (Wang *et al*., 2013).

In a similar vein, species with workers of both sexes (such as termites) will have an additional source of antagonistic selection acting on males, where divergent selection operates between males with helper and reproductive roles. This contrasts with Hymenoptera, where the function of a male is purely reproductive. The dynamic of antagonism becomes increasingly complicated to predict when so many polymorphisms are involved.

## V. CHALLENGES FOR UNDERSTANDING CASTE ANTAGONISM

### (1) Identifying phenotypes mediating antagonism

Measurement of caste antagonism presents a number of obstacles that are not present when measuring sexual antagonism. But as we discuss here, caste antagonism is empirically tractable and social systems provide novel ways to quantify role‐specific selection (Table 1).

In some social systems, it may be difficult to measure inter‐caste genetic correlations using quantitative genetics because of the confounding effects of the common environment shared by queens and workers. Although workers can often be maintained separately in the laboratory, normal colony functioning typically requires the castes to be kept together. Maternal and sib‐social effects can also strongly affect the phenotype, and must be considered when estimating the additive effects of genes (Linksvayer & Wade, 2005). In order to separate these effects it may be possible to transfer individuals between social groups (or nests) in cross‐fostering experiments (Linksvayer, 2006; Holman *et al*., 2013). Cross‐fostering can be conducted in the laboratory after colonies have been collected in the wild; a method used successfully in ants and bees (e.g. Linksvayer, 2006, 2007; Holman *et al*., 2013; Rittschof *et al*., 2015; Linksvayer, Fondrk & Page, 2009). Cross‐fostering is also possible in the wild in some social insects (e.g. Costanzi, Bagnères & Lorenzi, 2013). A multi‐generational breeding design (Lynch & Walsh, 1998) is another feasible option. Such controlled crosses are likely to be restricted to laboratory, or semi‐wild conditions, and are laborious but possible (e.g. Hayashi *et al*., 2007; Schwander & Keller, 2008; Frohschammer & Heinze, 2009).

Alternatively, an ‘inbred line’ approach with a social insect species that can tolerate inbreeding could be used. By estimating values of fitness‐related traits in queens and workers derived from a number of genetically homogeneous lines, one could estimate inter‐caste genetic correlations. Again, this method is likely to be confined to the laboratory. Its use could also be constrained by the study species, because in many social insects, inbreeding leads to diploidy and sterility in males. Despite this, there are some species where sib‐mating is common, such as the ant *Cardiocondyla obscurior* (Schrempf, Aron & Heinze, 2006). Inbreeding experiments have also been applied successfully in other social systems [e.g. *Pseudomyrmex ferruginea* ants (Mintzer & Vinson, 1985), *A. mellifera* bees (Clarke, Oldroyd & Hunt, 1992) and *Lasioglossum zephyrum* bees (Greenberg, 1979)]. Isofemale lines have been used analogously in non‐social insects to understand sexual antagonism (Berger *et al*., 2014; Punzalan, Delcourt & Rundle, 2014). An additional possibility would be to estimate genetic correlations between queen and worker traits in a pedigreed, wild population using an animal model approach (Kruuk, 2004). For example, microsatellite markers have been useful for inferring pedigrees in *Polistes dominula* (Queller *et al*., 2000) and *Cataglyphis* and *Pheidole* ants (Leniaud, Pearcy & Aron, 2013; Huang, Wheeler & Fjerdingstad, 2013). It might also be possible to use this method to measure the genetic correlation between queen fitness and the effect that a worker has on the productivity of a queen. This could be achieved by observing foraging behaviour in workers, for example.

When investigating inter‐sexual genetic correlations in the fruit fly *Drosophila melanogaster*, hemiclonal analysis has been an alternative to using genetically inbred lines. This allows the same haplotype to be expressed in both males and females in a heterozygous state, and also avoids the costs of homozygosity and selection associated with inbred lines [Table 1, and see Rice (1996), Chippindale *et al*. (2001) and Abbott & Morrow (2011)]. Hemiclonal analysis has enhanced our understanding of sexual antagonism because it allows for the additive genetic sex‐specific fitness effects of haplotypes to be quantified directly (Chippindale *et al*., 2001; Gibson *et al*., 2002; Pischedda & Chippindale, 2006; Long & Rice, 2007; Bedhomme *et al*., 2008; Innocenti & Morrow, 2010; Hesketh *et al*., 2013). Although this type of artificial genetic manipulation has so far been unique to *D. melanogaster*, the clonal sperm found in all hymenopteran social insects could be used in a similar way to disentangle the additive effects of genes on particular castes. Hymenopteran males are haploid and produce genetically identical sperm by mitosis, which means they are related to their daughters (future queens and workers) by 1.0, while their daughters are related to one another by 0.75. One way to explore the link between sexual antagonism and caste antagonism is to partition variance in offspring phenotype within and between colonies of the same and different patrilines. By knowing the relatedness between individuals it is possible to explore genetic effects on sex and caste‐specific fitness. Indeed, some social insect species normally exhibit multiple patrilines within colonies, whereby different workers share a maternal genome but inherit different paternal genes [e.g. *Acromyrmex* leaf‐cutting ants (Hughes *et al*., 2003), *Pogonomyrmex rugosus* ants (Schwander & Keller, 2008), *Pogonomyrmex badius* ants (Smith *et al*., 2008) and *A. mellifera* bees (Kraus, Gerecke & Moritz, 2011)]. In polyandrous study systems such as these, it is possible to infer the paternal line using microsatellite genotyping. A natural environmental control also exists, in that all patrilines within a colony share the same environmental cues. This method would allow us to ask whether particular alleles are correlated with differing performance when expressed in queens, workers and males. Kovacs *et al*. (2010) assigned queen and worker genotypes to patrilines in the social wasp *Vespula maculifrons* to explore inter‐caste phenotypic correlations in traits that are potentially related to fitness. They found a negative correlation across traits between caste dimorphism and the inter‐caste phenotypic correlation, suggesting that trait genetic architecture might be constraining independent adaptation within castes. However, the correlation was driven by a single trait, and in a separate study they showed that the same traits lacked a strong additive genetic basis, suggesting that caste antagonism is not currently involved (Kovacs *et al*., 2010).

### (2) Molecular insights

In addition to phenotypic studies, a thorough understanding of the evolutionary dynamics of caste antagonism ultimately requires identifying specific alleles that are maintained by caste‐specific selection and that underlie maladaptive trait variation within each caste. New research by Hill *et al*. (2017) identified specific alleles underlying sexually antagonistic fitness variation in *D. melanogaster*, and this research could also be replicated in social insects by correlating whole‐genome sequence variation with caste‐specific fitness effects. For both sexual antagonism and caste antagonism, more comprehensive molecular data sets (that incorporate gene expression, sequence variation and functional annotations) could also answer fundamental questions regarding the genetic constraints on antagonism resolution. For instance, are genes that are embroiled in caste antagonism also under pleiotropic or epistatic constraints preventing complete caste‐specific expression? This has been explored in relation to sexual antagonism by uncovering links between sex‐biased gene expression and proximate measures of pleiotropy, such as the tissue specificity and network connectivity of genes (Mank *et al*., 2008; Frings *et al*., 2012; Mank, 2016). A recent paper by Morandin *et al*. (2017) provides strong evidence that gene connectivity constrains caste‐biased expression in ants, setting the stage for further comparisons across other social insect taxa that would be useful to explore the ubiquity of these constraints further. Insight into the generality of genetic constraints on phenotype could also be gained by studying the molecular basis of other forms of phenotypic variation, such as alternative reproductive tactics within a sex.

The goal of understanding caste antagonism at the molecular level can be achieved most easily using organisms for which there is a history of genetic research. Since DNA sequence information was published (Honey Bee Genome Sequencing Consortium, 2006), *A. mellifera* has become a model organism for social insect genetics. More recently, however, as genome assembly methods have improved in efficiency and accessibility, sequence information has become available for various other social bee (Kocher *et al*., 2013), wasp (Toth *et al*., 2007; Ferreira *et al*., 2013), ant (Bonasio *et al*., 2010; Nygaard *et al*., 2011; Smith *et al*., 2011a,*b*; Suen *et al*., 2011; Wurm *et al*., 2011; Gadau *et al*., 2012; Oxley *et al*., 2014), and termite (Terrapon *et al*., 2014) species. The rich genetic information that is available for *D. melanogaster* (Del Valle Rodríguez, Didiano & Desplan, 2012) has similarly aided understanding of sexual antagonism (e.g. Rice & Chippindale, 2001; Innocenti & Morrow, 2010). These benefits will extend further to understanding caste antagonism because hom ologs of genes under caste antagonism in social insects can be scanned for in *D. melanogaster*, possibly aiding their functional annotation. Many genes have functions that are conserved between *Drosophila* and social insects, such as those with reproductive and foraging functions (Toth & Robinson, 2007) that might mediate traits involved in caste antagonism.

### (3) Social group dynamics

As well as being of interest in its own right, understanding the involvement of specific traits in caste antagonism could help to answer general questions about social group dynamics. One example is exploring caste antagonism over egg production and its effect on the partitioning of reproduction between the members of a social group (‘reproductive skew’; Nonacs & Hager, 2011). For example, there is a lack of among‐colony variation in reproductive skew within species of primitively eusocial wasps, despite considerable variation in factors predicted to affect skew in strategic models (Field & Cant, 2009). This might result from inter‐caste genetic correlations for traits affecting dominance and within‐colony competition for reproduction, such that alleles resulting in more‐fecund queens also result in more‐fecund workers, with skew remaining unchanged.

## VI. DIVERSITY OF SYSTEMS FOR EMPIRICAL TESTS OF CASTE ANTAGONISM

### (1) Social complexity and caste specialisation

An extraordinarily diverse collection of social insect species exists that could provide different insights into caste antagonism (e.g. Fig. 1). For example, species differ greatly in social complexity, from termites and ants (Fig. 1) with worker castes that often completely forgo direct reproduction and have high functional specialisation (Anderson & McShea, 2001; Eggleton, 2011), to primitively eusocial bees and wasps that lack morphological castes altogether [e.g. *Polistes* paper wasps (Reeve, 1991; Fig. 1) and halictid bees (Danforth, 2002)]. Some species are even socially polymorphic at the population level, with separate eusocial and non‐social populations [e.g. the sweat bees *Halictus rubicundus* (Soucy & Danforth, 2002; Field *et al*., 2010) and *Lasioglossum calceatum* (Sakagami & Munakata, 1972; Davison & Field, 2016)]. Species (or populations) with more functionally specialised castes are likely to show more extensive patterns of caste‐specific gene regulation and higher levels of existing caste antagonism.

### (2) Termites

Termites are of particular interest for caste antagonism research as they evolved sociality independently of the Hymenoptera. A factor that is likely to change the dynamic of caste antagonism in termites is the presence of sex chromosomes as opposed to haplodiploid sex determination. This difference in chromosomal structure has two main consequences: (*i*) the movement of genes to sex chromosomes offers a potential resolution to intralocus sex and caste antagonism, and (*ii*) diploidy in males reduces the exposure of alleles to selection, which can alter the evolutionary trajectory of traits shared between sexes and castes. Levels of sexual dimorphism are also low in termites relative to social Hymenoptera (Boomsma *et al*., 2005), and workers of both sexes exist, in contrast with exclusively female workers in Hymenoptera. In some termite species, there appears to be a link between sex‐ and caste‐determination. In *Reticulitermes speratus* a sex‐linked locus with two alleles is responsible for caste determination, where the same allele that causes males to develop into reproductives also causes females to develop into workers; whereas the other allele causes males to develop into workers and females to develop into reproductives (Hayashi *et al*., 2007). In a different study, it was suggested that termite species with greater levels of sexual size dimorphism tend to have workers of a single sex that are more specialised (Bourguignon, Yoshinobu & Miura, 2012). These examples suggest that sexual dimorphism might have enabled functional specialisation of worker castes, as predicted if mechanisms to resolve one conflict act to mitigate the other. A comparison with the more sexually dimorphic Hymenoptera could be useful for testing this further.

### (3) Unusual genetic systems

Some social insects have unusual genetic systems, in which we hypothesise that caste antagonism should shape genomic architecture in different ways. In at least three ant species, queens are produced asexually and workers sexually, while males are genetic clones of the queen's mate (Wenseleers & Van Oystaeyen, 2011). Provided that workers are sterile and queens are never produced sexually, this means that each species is composed of two genetically isolated lineages: one that is present in queens and workers, the other in males and workers. We predict that if the male phenotype is closer to the worker optimum, worker‐beneficial alleles should be more prevalent in the latter lineage and queen‐beneficial ones in the former, although the evolutionary outcome will likely depend on the interplay between sexual antagonism and caste antagonism. Strong genetic caste determination occurs in other social insects, such that crosses between genetically divergent lineages produce workers, while within‐lineage crosses produce queens (Schwander & Keller, 2008). We suspect that this mode of caste determinism may also interact with caste antagonism, because it should result in workers having greater genome‐wide heterozygosity than queens. Selection on recessive alleles with caste‐specific fitness effects will therefore be more effective in queens, potentially causing shared phenotypes to be closer to the queen optimum relative to species without these unusual genetic systems.

## VII. GENERAL IMPLICATIONS OF CASTE ANTAGONISM

The mechanisms by which genetic variation is maintained within populations have long interested evolutionary biologists (Mather, 1955; Charlesworth, 1987; Andersson & Iwasa, 1996; Rowe & Houle, 1996; Lynch & Walsh, 1998; Kingsolver *et al*., 2001; Haag‐Liautard *et al*., 2007; Trotter & Spencer, 2007). The idea that correlated selection between the sexes could maintain fitness variation has also been recognised for some time (Kidwell *et al*., 1977; Rice, 1984), and analogous mechanisms could operate as a consequence of correlated selection between other sets of individuals that have different phenotypic optima and a shared genetic architecture. Thus, caste antagonism should act similarly to maintain maladaptive genetic variation within castes. The maintenance of genetic variation that prevents caste‐specific adaptation could impact trait evolution and influence a broad range of biological phenomena. Below, we briefly discuss four possible areas of current interest: caste ratios, personality traits, ageing and disease.

### (1) Caste ratio

First, caste antagonism has the potential to explain differences between colonies in the caste ratio, i.e. the ratio of queens to workers produced by the colony. This could involve a queen adjusting offspring phenotype (caste) depending on whether her genotype would produce offspring best suited to a queen or worker role. In a similar way, sex ratio adjustment in response to sexual antagonism – where females might alter the number of sons *versus* daughters produced depending on whether the offspring will carry male‐ or female‐beneficial alleles – has been proposed to occur in dioecious species (Blackburn, Albert & Otto, 2010). In social insects, caste ratio adjustment would require some level of caste‐fate control by queens, through nutritional (Hunt, 1991, 2007; Anderson, Linksvayer & Smith, 2008; Kucharski *et al*., 2008), pheromonal (Vargo & Passera, 1992; Matsuura *et al*., 2010) and/or hormonal (Schwander *et al*., 2008) mechanisms. It would also require the presence of a cue by which a queen could determine the genotype of herself or her mating partner with respect to queen‐ *versus* worker‐beneficial alleles. Colonies carrying an excess of worker‐beneficial alleles at antagonistic loci might also benefit from producing males over queens, although here it is difficult to make reliable predictions without explicit theoretical models, due to the complex frequency‐dependent selection that can result from facultative sex ratio adjustment. An example of caste ratio adjustment that is dependent on a perceived cue is where shifts in caste occur in *Pheidole pallidula* ants (due to nutritional provisioning) when threats from competitors are detected (Passera *et al*., 1996). Alternatively, it could be that caste ratio differences arise because queens with some genotypes produce more viable offspring of one caste than the other.

### (2) Personality

Caste antagonism could be responsible for maintaining variation in ‘personality’ – i.e. repeatable inter‐individual differences in behaviour – which have been documented in social insects (e.g. Jandt *et al*., 2014). If we suppose that certain heritable behaviours, for example aggression, foraging rate, and activity level, are under caste‐specific selection, then it follows that caste antagonism may elevate genetic variance in these traits and thereby lead to inter‐individual differences within a caste. There is some evidence that behavioural traits in social insects have a heritable genetic basis (Van Oers *et al*., 2005; Penke, Denissen & Miller, 2007; Van Oers & Mueller, 2010), and caste antagonistic selection on behaviour seems probable but has yet to be empirically demonstrated.

### (3) Ageing

Caste antagonism is also likely to maintain alleles that influence processes of ageing (Adler & Bonduriansky, 2014). For example, queens and workers (and males) are often highly dimorphic in lifespan (Carey, 2001). Extreme cases are provided by certain ants, where queens can live up to 30 times longer than their worker offspring (Holldobler & Wilson, 1990). This divergent selection on lifespan stems from differences in extrinsic mortality risk, such as predation, which is greater for workers than queens (Keller & Genoud, 1997). Dimorphism in lifespan and senescence could reflect either past caste antagonism that has been resolved through caste‐specific expression, or partially resolved but on‐going antagonism. There is already evidence that sexual antagonism over lifespan can occur in insects, despite the evolution of sexual dimorphism. Lewis, Wedell & Hunt (2011) demonstrated sexually antagonistic selection as well as genetic constraints on longevity in the moth *Plodia interpunctella*, which together resulted in the sexes being displaced from their respective phenotypic optima. Berg & Maklakov (2012) conducted artificial selection on longevity in the beetle *Callosobruchus maculatus*, uncovering an inter‐sexual genetic correlation coupled with a negative correlation for fitness between the sexes. Although this is potentially a widespread source of antagonism in social insects too, empirical investigation is required.

### (4) Disease

A fourth possibility is that caste antagonism could contribute to the occurrence of disease. An example is the disease outbreaks that occur in commercial honeybee populations (Cox‐Foster & Van Engelsdorp, 2009), which are of considerable public and scientific interest. In sexual antagonism research, immune function has been associated with antagonism (Mckean & Nunney, 2005; Rolff, Armitage & Coltman, 2005; Svensson *et al*., 2009; Calsbeek & Bonneaud, 2008; Innocenti & Morrow, 2010) and it is expected that disease, or alleles influencing disease susceptibility, are also maintained in human populations by sex‐specific or sexually antagonistic selection (Gilks, Abbott & Morrow, 2014). In social insects, the immunity of queens and workers should be under divergent selection because queens might require higher immunity to increase lifespan and sustain fecundity for long periods, whereas workers do not. Alternatively, workers might require higher immunity, since they tend to leave the nest more often. In line with this, caste dimorphism in genes influencing immunity has been demonstrated in social insects (e.g. Pereboom *et al*., 2005; Gräff *et al*., 2007; Grozinger *et al*., 2007). There is also heritable genetic variation within bee populations for susceptibility to parasites and infection (reviewed in Grozinger & Robinson, 2015), but because inter‐caste genetic correlations have not yet been measured it is unclear whether this variation is maintained by caste antagonism.

## VIII. CONCLUSIONS

(1) Caste antagonism is a conflict between social castes over gene expression. The conflict arises because castes share a genome but have divergent phenotypic optima. The fittest allele at a given locus will then often not be the same across the different castes. Given the varied and contrasting roles that queens and workers occupy across social insect taxa, this conflict is likely to be widespread in nature. Despite this prediction, caste antagonism is a greatly understudied field of research in comparison with sexual antagonism, an analogous conflict between males and females, which is now thought to be ubiquitous.

(2) Evidence for a positive inter‐caste correlation for fecundity suggests that queens and workers are constrained from reaching their phenotypic optima in ants and bees. Quantifying other physiological and behavioural phenotypes associated with this correlation, such as how it affects worker productivity, would be useful for further exploration of the potential for caste antagonism. Additionally, very few studies have estimated inter‐caste correlation, and it is not clear if it is similarly strong throughout the social insects.

(3) An increasing number of studies of the molecular biology of social insects have revealed dimorphism in gene expression between queens and workers, suggesting that caste antagonism has at least been partially resolved. There is mixed evidence for the involvement of gene duplication, gene splicing, and methylation in caste differentiation, paralleling routes involved in the evolution of sexual dimorphism. In contrast to intralocus sexual conflict, caste antagonism cannot be resolved by the movement of genes to sex chromosomes, since these are absent in social Hymenoptera.

(4) Given that social taxa have different sexes as well as different castes, antagonism over gene expression is likely to extend further than just the conflict that exists between castes – creating a three‐ (or more) way conflict. Although unexplored so far, this could alter the dynamics of the conflict and its resolution in unexpected ways. The net evolution of any trait will be dependent on the specific selection pressures on each individual, and it is unlikely that evolution will optimise all phenotypes. The outcome will also be affected by haplodiploidy, where the efficiency of selection will be greater in males. In the same vein, relatedness structure and co‐variation between direct and indirect genetic effects that occur within social groups will add an additional layer of complexity. Moreover, the conflict is likely to extend beyond a three‐way interaction, as it will be affected by selection from breeders that have different reproductive tactics, as well as workers that take on different roles within the colony.

(5) Detecting caste antagonism is empirically tractable, and our understanding of it could be aided by borrowing concepts from sexual antagonism research, but also through the use of empirical methods that are unique to social systems. For example, genetic and environmental effects on caste fitness‐related traits can be disentangled through quantitative genetic breeding designs and by investigating wild populations where pedigrees are known. The clonal sperm of hymenopteran males might also be exploited to explore the additive effects of genes on caste‐specific fitness (see Table 1). Additionally, a huge variety of insect social systems exist that can offer insights into caste antagonism, such as those differing in level of social complexity and caste specialisation.

(6) Caste antagonism can help to explain why we see variation for fitness‐related traits within castes in natural populations. This is expected to impact trait evolution, with far‐reaching effects on biological processes, including altering caste ratios within social groups, and creating the genetic variation underlying personality traits, lifespan and disease. By exploring caste antagonism in more detail, our understanding of adaptive processes of evolution could be greatly improved.

(7) Sexual antagonism research provides inspiration and the initial building blocks for outstanding questions in caste antagonism research. There is ample opportunity for these two research streams to complement one another, since they involve analogous conflicts that lend themselves to contrasting experimental approaches. Understanding caste antagonism also has the potential to shed new light on questions about the evolution of sociality, such as the genetic mechanisms that promote and prevent caste differentiation, and the processes underlying reproductive skew.
